# A comparison of geometric- and regression-based mobile gaze-tracking

**DOI:** 10.3389/fnhum.2014.00200

**Published:** 2014-04-08

**Authors:** Björn Browatzki, Heinrich H. Bülthoff, Lewis L. Chuang

**Affiliations:** ^1^Department of Perception, Cognition and Action, Max Planck Institute for Biological CyberneticsTübingen, Germany; ^2^Department of Brain and Cognitive Engineering, Korea UniversitySeoul, South Korea

**Keywords:** calibration method, gaze measurement, eye tracking, eye movement, active vision, gaussian processes

## Abstract

Video-based gaze-tracking systems are typically restricted in terms of their effective tracking space. This constraint limits the use of eyetrackers in studying mobile human behavior. Here, we compare two possible approaches for estimating the gaze of participants who are free to walk in a large space whilst looking at different regions of a large display. Geometrically, we linearly combined eye-in-head rotations and head-in-world coordinates to derive a gaze vector and its intersection with a planar display, by relying on the use of a head-mounted eyetracker and body-motion tracker. Alternatively, we employed Gaussian process regression to estimate the gaze intersection directly from the input data itself. Our evaluation of both methods indicates that a regression approach can deliver comparable results to a geometric approach. The regression approach is favored, given that it has the potential for further optimization, provides confidence bounds for its gaze estimates and offers greater flexibility in its implementation. Open-source software for the methods reported here is also provided for user implementation.

## 1. Introduction

Using gaze-tracking methods, it is possible to record where someone is looking on a visual display. Such methods facilitate the continuous observation of natural behavior, such as reading or visual search. In the context of electroencephalography (EEG) research, it allows neural activity to be co-registered with a visual stimulus that the participant chose to fixate (Baccino and Manunta, 2005; Jagla et al., 2007).

Unfortunately, accurate gaze-tracking often requires the participant's head and body movements to be restrained, for example, with a head-rest. As a consequence, the eye's position is fixed in a global reference frame and accurate gaze-tracking can be achieved by tracking only the rotations of the eye. This can be achieved either by tracking the induction current of a coil that is placed on the eye itself (Robinson, 1963; Collewijn et al., 1985) or with video-based eye-trackers, which utilize either head-mounted or long-range cameras to monitor characteristic visual features of the eye (i.e., pupil, corneal reflection). Video-based methods are non-invasive and are, thus, more comfortable to the user and suitable for studying natural behavior for longer test sessions. During calibration, visual stimuli (e.g., 0.5° radius annulus) are presented at extrema points on the display for fixation. By interpolating between the pupil position in the eye-tracker's camera image, it is possible to infer the observer's point of regard (POR) between these extreme screen positions. If the physical distance of the observer's eyes to these calibrated points are known, it is possible to infer the vertical and horizontal rotations of the observer's eye in a head-centered coordinate system (Nakayama, 1974; Moore et al., 1996).

If the observer's head pose is known (i.e., combined position and orientation), this geometric approach can be extended to compute gaze without restraining head movements (Epelboim et al., 1995; Johnson et al., 2007; Ronsse et al., 2007). Continuous measures of a user's head pose can be achieved with motion tracking systems. Such systems range from off-the-shelf markerless motion-tracking systems (e.g., Microsoft's Kinect) to those that track well-placed infra-red reflective markers on the user's body with a high level of precision (e.g., Vicon Motion Systems). The critical step lies in deriving the transformation matrix that expresses the eye model, which is calibrated in an eye-centered reference frame, in terms of the global reference frame that the user and task relevant objects share (see section 2.2). This defines a line-of-sight. Namely, a gaze vector that consists of the eye's origin and direction. If an accurate model of the display (and/or other real-world objects) in the same global reference frame is known, intersections between the current line-of-sight and the screen coordinates of the display can be easily computed.

There are several limitations to this geometric approach. On the one hand, it requires an accurate model of the display as well as of the obsever's eye. Such models are often represented as idealized geometric objects and their interdependence must be explicitly stated as linear algebraic formulations. These formulations do not consider intrinsic error through non-linearities and inaccurate measurements during the calibration phase. For example, there might be small but systematic displacements of head-mounted eye-tracking cameras due to tension of the forehead muscles when fixating peripheral targets (e.g., >15°). This would cause non-linearities in the eye-model that are rarely accounted for. Finally, the geometric approach assumes that the vector of the user's gaze accurately intersects with the POR during the calibration phase. In reality, gaze stability is likely to vary across individuals and different activities, regardless of compensatory eye movements (e.g., vestibular ocular reflex; Medendorp et al., 2002). Even if gaze fixation can be assumed to be perfectly stable by minimizing head and body movements during calibration, this may not be the case during testing. Altogether, these small residual errors could accumulate and result in a significant combined error. In fact, the calibration accuracy of the eye-tracker is especially critical in a geometric-based system since this is the only aspect that can be controlled by the experimenter during data-collection. Therefore, it is often repeated until an acceptable level of error is achieved. If this is not possible, the experiment is aborted.

In contrast to the geometric approach, a purely data-driven regression approach could enable data from the motion- and eye-tracker to be directly mapped to the desired coordinates for POR. For example, the screen coordinates of the display(s) or object. This mapping can be inferred from training data without the need for any domain specific knowledge. In addition, system error or unanticipated behavioral singularities need not be explicitly specified as they will be implicitly incorporated in the model. Such an approach does not attempt to geometrically reconstruct the line-of-sight. However, data-driven methods suffer from the fact that outputs are highly dependent on the training data. This means that they can only be as accurate as the data provided during calibration. And, they require behavior in the calibration phase to resemble expected behavior during testing. This may require an inordinate amount of training data, translating into a impractically long calibration phase. Nonetheless, it grants the experimenter the flexibility (and responsibility) of designing the calibration task so as to solicit looking behavior that best generalizes to the test conditions. Finally, a regression method will not only provide an estimate of the POR, but an associated confidence level as well. This can be obtained prior to experimentation and would determine if more data is required for further calibration. It can also be used to filter out unreliable PORs from the test data.

The purpose of the current work is threefold. First, it provides a comparison of a geometric and a regression approach to mobile gaze-tracking. To evaluate both methods, we adopted a calibration–validation protocol—a procedure that is common to most commercial eye-tracking systems. Data from a single user is first processed with one calibration method and then validated in terms of its accuracy in determining the user's gaze on known PORs. Therefore, our reported results should provide readers with a practical intuition of the data quality that can be expected when using either a geometric or a regression method. Previous reports on mobile gaze-tracking restricted their analyses to standing participants with unrestrained head movements (e.g., Ronsse et al., 2007; Cesqui et al., 2013). Here, we included a previously unreported condition that required our participants to walk freely. Second, we address how the procedure for collecting calibration data can influence the validation accuracy of either method. For this purpose, we collected datasets in two situations. Participants either fixated an unpredictable sequence of static markers (cf., Johnson et al., 2007) or pursued a moving marker (cf., Cesqui et al., 2013). Our algorithms were trained on either type of dataset and validated on the same or different type of dataset. Third, we provide the approaches reported in this paper as an open-source software toolbox to allow other researchers to implement the methods reported here in their own test environments and adapt them to their specific needs. Some variations of the geometric approach have been reported before (e.g., Epelboim et al., 1995; Johnson et al., 2007; Ronsse et al., 2007; Cesqui et al., 2013). Our implementation represents a general version of these methods and does not rely on specific equipment or assumptions. For example, we do not assume a particular geometric model of user's eye and head. It should be noted that our implementation is only intended for the retrieval of a mobile user's POR. It does not offer the level of spatial and temporal precision required for the study of gaze kinematics. For this, a scleral search-coil method should be employed instead.

This paper is organized as follows. Section 2 provides a systematic description of the geometric and the regression methods that we implemented for mobile gaze-tracking. Excellent textbooks are available that provide a comprehensive coverage of the basics of eye-tracking methodology as well as details of various implementations, and discussion of their relevance to behavioral research (i.e., Duchowski, 2007; Holmqvist et al., 2011). Section 2.4 reports a side-by-side evaluation of our geometric and regression methods. Three levels of user mobility were tested: (a) head-fixed, (b) head-free, (c) walking. The evaluations also explored instances where our regression method fared poorly, so as to highlight the limitations of this approach. We conclude by discussing the strengths and limitations of using either approach.

## 2. Materials and methods

### 2.1. Implementation and system overview

In this section, we describe a geometric and a regression-based method for mobile gaze-tracking. These are publicly available as open-source software for mobile unrestrained gaze-tracking (*MUG*; https://bitbucket.org/browatbn/mug). Both methods require a motion tracking system and a head-mounted video eye-tracker for input data (**h, p**). The motion-tracking system provides the position and orientation of the user's head in a world coordinate system, which is collectively referred to as its pose, **h** = (*h*_*x*_, *h*_*y*_, *h*_*z*_, *h*_ϕ_, *h*_θ_, *h*_ψ_). The eye-tracker provides the 2-dimensional position of the user's pupil in the camera image, **p** = (*p*_*x*_, *p*_*y*_). The output of both methods is the user's POR, given as the horizontal and vertical coordinates of our screen model, (*u, v*). Although we assumed a planar surface for the current evaluation, this could be replaced by models with other display configurations (e.g., a curved screen), without modification of the core calibration algorithms *per se*. To the best of our knowledge, our methods do not depend on any proprietary algorithms of the chosen hardware systems, ensuring the generalizability of our methods to other hardware systems.

**Figure 1:**
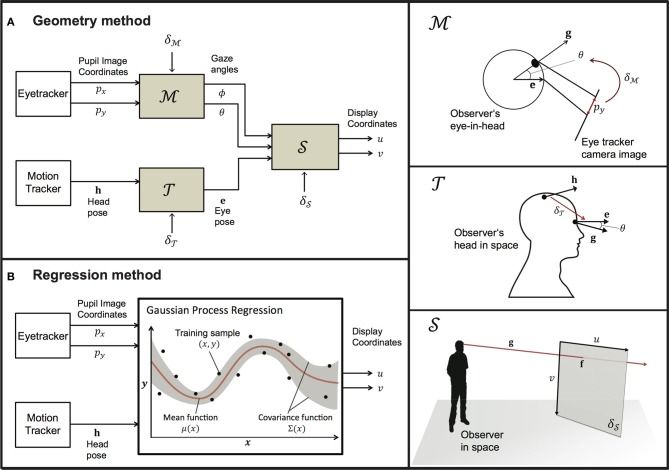
**A flowchart representation of the geometric (left panel; A) and regression (left panel; B) approach**. Both approaches map data from the eye- and motion-tracking systems to PORs within the display coordinate system. The right panel describes the models that underlie the geometric method and their associated parameters.

Sections 2.2 and 2.3 provide an overview of the algorithms on which our geometric and regression implementations are based. Figure 1 provides a flowchart of the underlying processes of each method. Our geometric implementation operates by deriving the optimal parameters for a head-to-eye transform model (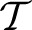), an eye-in-head model (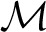) and a screen model (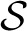) from eye- and motion-tracker data that is collected during the calibration phase. In section 2.2, we describe these three models separately, before addressing how these models are simultaneously calibrated on the input data of a mobile user from the motion- and eye-tracker. Our regression-based implementation relies on Gaussian process regression, which estimates the best fitting multi-variate Gaussian distribution that directly maps input data from the motion-tracking system and the eye-tracker to screen coordinates in the display.

### 2.2. Geometric approach

The geometric approach treats gaze as a vector in space that is jointly defined by the position and orientation of the eye in space **e** and the eye's rotation about its horizontal and vertical axes, ϕ and θ, respectively. However, a video-based eye-tracker can only provide estimates of the eye's rotations about its center. In addition, a motion-tracking system can only provide the position and orientation of the tracked markers, which have an unknown position and orientation offset to the center of the eye depending on their placement on the user's head. Thus, calibration consists of deriving the optimal parameters for a head-eye-transformation model (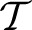) and an eye-in-head model (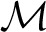), based on input data that is collected from the eye- and motion-tracking system when the user is fixating known positions in space. These fixations are typically elicited by requiring the user to fixate a sequence of annuli on a visual display. If unknown, a physical representation of the visual display 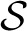 can also be estimated from the input data, given the shape parameters of the visual display and the assumption that the user is accurately fixating the presented stimulus.

#### 2.2.1. Head-eye-transform model

The head-eye-transform model 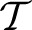 derives the eye's pose in the world coordinate system **e** from the motion-tracking data, which provides an estimate of the head's pose in the world coordinate system **h**. This transformation is affected by the user's anthropomorphic characteristics as well as the placement of the tracking markers on the user's head. These parameters δ_
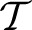
_ have to be estimated from calibration data.

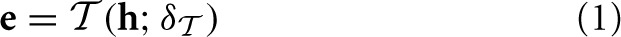



Given that the eye is located at a fixed position (*x*_
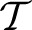
_, *y*_
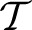
_, *z*_
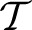
_) relative to the position of the motion-tracking markers, which are attached to the user's head (*h*_*x*_, *h*_*y*_, *h*_*z*_), and has a orientation of (θ_
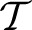
_, ϕ_
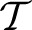
_, ψ_
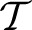
_), δ_
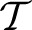
_ defines the affine transformation from the head-centered reference frame to the user's eye-centered reference frame:

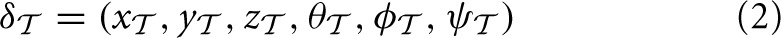



The eye position (*e*_*x*_, *e*_*y*_, *e*_*z*_)^*T*^ is defined by a rotation of the eye's position offset (*x*_
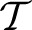
_, *y*_
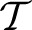
_, *z*_
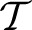
_) around the tracked head position (*h*_*x*_, *h*_*y*_, *h*_*z*_). The superscript *T* is used to indicate the transpose of a matrix or vector. This rotation is specified by the head's orientation, expressed as a rotation matrix *R*_**h**_:

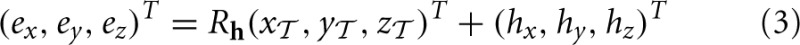



We express the eye orientation in the form of a rotation matrix *R*_**e**_. This is calculated by multiplying the current head orientation matrix *R*_**h**_ with the rotation matrix *R*_
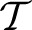
_, which is defined by the rotational components (θ_
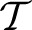
_, ϕ_
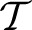
_, ψ_
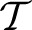
_) of the head-to-eye transformation:

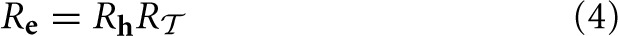



Thus, *R*_**e**_ represents the transformation from the tracked head orientation to the orientation of the eye-centered reference frame in the world coordinate system.

#### 2.2.2. Eye model

The eye-tracking camera captures a pupil image and from this an eye model 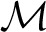 is necessary to map the pupil's centroid position in the camera image, *p*_*x*_, *p*_*y*_, to the rotations of the eye, ϕ, θ, about its center:

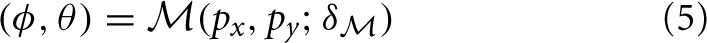



This mapping is determined by position, size and orientation of the eye with respect to the camera's image plane. The parameters that are necessary to calculate this mapping are denoted as δ_
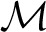
_ and depends on the assumed relationship between the recorded eye and the obtained camera image. For example, an established model by Moore et al. (1996) assumes the pupil to be the center of a plane section (i.e., the iris) that is located on a perfect sphere at a fixed distance from the eye's centroid. Here, the pupil location in the camera image is treated as a perspective projection of the eye onto the image plane (see Cesqui et al., 2013 for a treatment of the pupil image as an orthographic projection instead).

In the current work, we assumed a linear correlation between the pupil's image positions and their corresponding rotation angles of the eye. This is expressed as linear models in Equations (6, 7).

(6) $$\phi =m_{\phi }p_{x}+b_{\phi },$$

(7) $$\theta =m_{\theta }p_{y}+b_{\theta },$$

The parameters *m* and *b* are fitted to eye-tracking data obtained in a calibration procedure. This is explained in more detail in section 2.2.4. This approximation is motivated by computational efficiency and is a reasonable assumption, if δ_
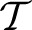
_ is chosen appropriately. Doing so allows us to compute the model parameters *m* and *b* with a simple linear regression. We implemented the more complex model of Moore et al. (1996) but did not find a significant difference between the two eye models with respect to our evaluations. Both models are available in our software implementation.

#### 2.2.3. Screen model

The screen model 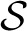 provides a mapping between the display's 2D screen coordinates and the three-dimensional Cartesian coordinates of the same display.

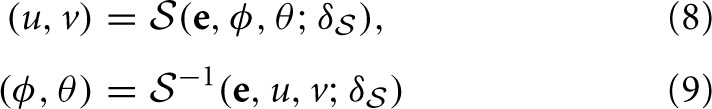



Assumptions about the display size, position, orientation, curvature, etc. are collectively expressed as δ_
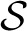
_. The screen model relies on these parameters and the outputs of the head-eye-transform model (i.e., **e**) and the eye model (i.e., ϕ, θ) to estimate POR in terms of the display's horizontal and vertical screen coordinates (i.e., *u, v*). Conversely, the inverse of the screen model allows us to estimate the rotation angles of our eye-model, given the current eye pose and screen coordinates of the user's POR. This is a necessary step in the calibration algorithm as it allows the rotation angles of the eye to be estimated from a known POR on the display, such as when the user is fixating a specified calibration stimulus.

For current purposes, we assume that the screen is a planar surface that is defined by a center point **c**, a normal vector **n**, a metric width and height *s*_*x*_, *s*_*y*_, as well as a corresponding display resolution of *s*_*u*_ × *s*_*v*_ in pixels. Thus, we define δ_
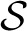
_ as

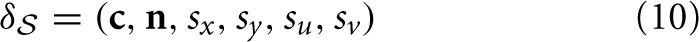



From a known eye pose, **e**, a gaze vector onto the screen can be calculated by multiplying the rotation matrices of the eye's orientation in space (Equation 4) and its rotations about its center:

(11) $$g=R_{e}R_{\phi }R_{\theta }{\left(1,0,0\right)}^{T}$$

The 3D intersection point, **f**, of this gaze vector, **g**, and the display screen is determined. This constitutes the user's POR. With this, the current POR can be computed in terms of screen coordinates (*u, v*) by an interpolation that is based on the display screen's dimensions *s*_*x*_ × *s*_*y*_ and its pixel resolution *s*_*u*_ × *s*_*v*_.

#### 2.2.4. Calibration algorithm

In our geometric implementation, calibration works by requiring the participant to fixate on known positions on the display surface. Given the known PORs during calibration and the input data provided by the eye- and motion-tracking systems, our algorithm seeks to estimate the optimal values for the free parameters δ_
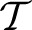
_, δ_
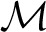
_, and δ_
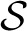
_. The screen model parameters δ_
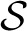
_ are only dependent on the display surface of the experiment and not the user. Thus, it only needs to be determined once. The parameters of the head-eye-transform model, δ_
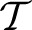
_, and eye model, δ_
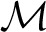
_, are user-specific and must be calculated for each individual participant.

This process is comparable to the standard calibration procedure of eye-trackers, whereby the head-fixed user is required to fixate on a sequence of annuli on the visual display. The sequence usually samples from a 3 by 3 grid that is centered and aligned to the display's boundaries. Based on the pupil's position on the camera image for each pre-determined POR, PORs on other regions of the screen within this grid can be estimated by interpolation.

For a mobile user, walking and head movements result in a changing head pose. These extra degrees of freedom must be accounted for in the calibration process. This can be achieved by performing the eye-tracker's calibration first, separately from the calibration of the head-eye-transform model (e.g., Johnson et al., 2007). Alternatively, one could optimize all the free parameters in one combined calibration process—for example, by requiring the user to fixate a known location in space while moving in a way that samples the range of possible head and body movements (Ronsse et al., 2007).

Like Ronsse et al. (2007), we optimize the free parameters of our models (i.e., 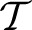, 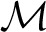, and 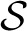) simultaneously. Unlike Ronsse et al. (2007), we do not require the user to perform any specific movement behavior. Instead, we presented a moving display stimulus that the user had to fixate, while moving his head and body according to the mobility that was permitted to him as per the experimenter's instructions. Details of our stimulus and mobility instructions are given in section 2.4. In this way, each participant provides a sample of calibration data that reflects his natural eye- and head-movements whilst fixating many PORs that cover a large area of the visual display.

Calibration data consists of a set of *n* input/output pairs 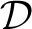 = {*x, y*}_*n*_ across the time of the calibrated session. The input data *x* = (**h**, *p*_*x*_, *p*_*y*_) gives the user's head and eye configuration and the output *y* = (*u, v*) represents the screen-coordinates of the calibration stimulus. The former is provided by the motion-and eye-tracking system while the latter is (randomly) determined by the experimental control script.

Since the screen configuration is independent of the current user, the calibration process can incorporate multiple datasets, acquired from different users. We denote the combined training corpus as 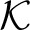 = (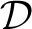
_1_, …, 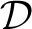
_*K*_). Screen model parameters δ_*S*_ are obtained by minimizing a cost function *t*_
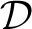
_ over each 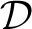. This can be stated as:

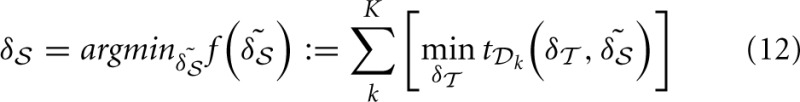



Function *t*_
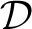
_ returns the difference between estimated PORs of the algorithm and the true PORs, based on the current parameters δ_
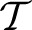
_ and δ_
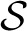
_ on the dataset 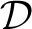 of a given user:

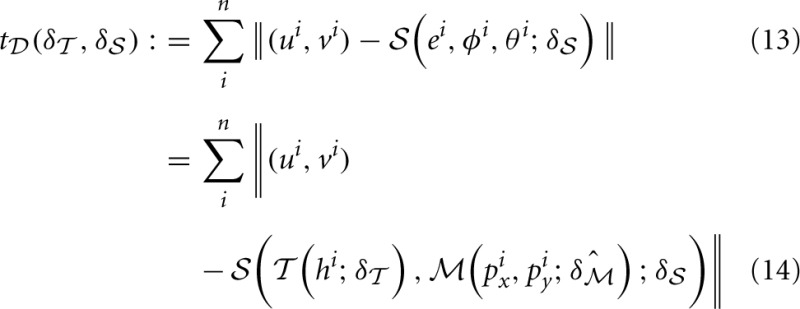



In evaluating Equation (13), an eye model 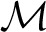 is used to estimate eye-rotation angles (ϕ, θ) based on the pupil's position in the camera image (*p*_*x*_, *p*_*y*_). To optimize its parameters (i.e., δ^^^_
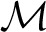
_), we carry out a minimization of the error between eye-rotation angles estimated based only on δ_
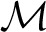
_ and eye-rotation angles geometrically calculated from the current parameterization of the screen model and head-eye-transform model (i.e., δ_
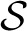
_ and δ_
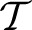
_):

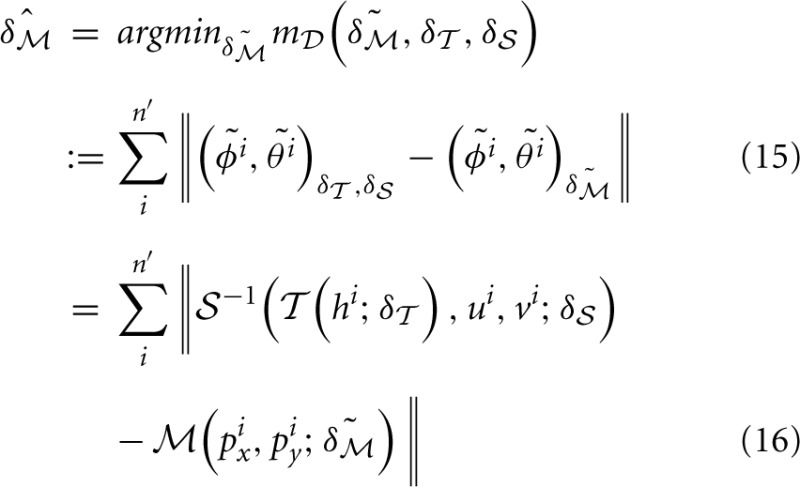



To increase computational efficiency, this optimization can be carried out on a subset 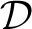
′ of 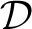, with *n*′ = |
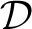
′| ≤ |
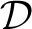
|.

After the screen model has been determined, the user specific parameters of the head-eye-transform model and eye models (i.e., δ_
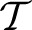
_, δ_
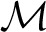
_) need to be optimized for each user. The head-eye transformation coefficients are determined by minimizing Equation (13) on user data 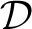:

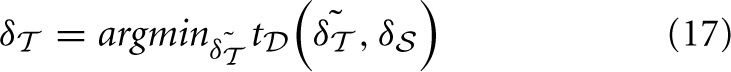



Likewise, we derive δ_
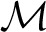
_ by evaluating Equation (15):

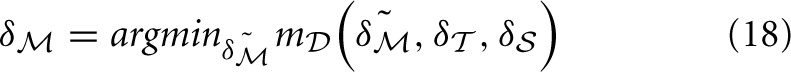



When computing (Equation 17), values for δ_
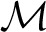
_ are acquired as well. Similarly, we find δ_
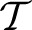
_ and δ_
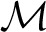
_ when evaluating (Equation 12). However, once the parameters for any given model are determined, the implementation of the other models can be further modified. As an example, the optimization of the screen model may be based on a simple eye model, while a more complex (but computationally intensive) eye model could be employed as the actual representation for subsequent experiments.

The minimizations of Equations (12), (17), and (18) can be accomplished employing any non-linear optimization method. We rely on the dlib implementation (King, 2009) of the BOBYQA algorithm (Powell, 2009).

### 2.3. Regression approach

In contrast to a geometric approach, a regression approach operates by predicting output data directly from a set of input data, without specifying the explicit relationships between them. It does not attempt to derive the user's line-of-sight (i.e., gaze) and its intersection with the display. Instead, it infers the relationship between the input and output values from a training set or calibration sample and then generalizes novel input data to a POR.

#### 2.3.1. Gaussian process regression

The Gaussian process regression (GPR) is a non-linear modeling technique that is able to predict the output *y* = *f*(x_*_) of a data point x_*_ based on a set of observations 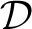 = (*x*_*i*_, *y*_*i*_)^*N*^_*i* = 1_. Rasmussen and Williams (2005) provides a thorough introduction to the method and its applications. In GPR, the underlying function *f* is represented as a Gaussian process that is defined by a multi-variate Gaussian distribution with a mean function μ and a covariance function Σ

(19) $$\mu_{*}=K_{*}K^{-1}y$$

(20) $$\Sigma_{*}=K_{**}-K_{*}K^{-1}K_{*}^{T}$$

where K_*_ = [*k*(*x*_1_, x_*_), …, *k*(*x*_*n*_, x_*_)] determines the covariance vector between training data and current test input. Similarly, K_**_ = *k*(x_*_, x_*_) and *K* is defined as the *n* × *n* covariance matrix of the training inputs such that

(21) $$K_{ij}=k(x_{i},x_{j})+\sigma_{n}^{2}\delta_{ij},$$

where δ_*ij*_ represents the Kronecker delta function. There are numerous possibilities for specifying the kernel function *k*(*x*_*i*_, *x*_*j*_). In our implementation, we employ the automatic relevance determination (ARD) kernel:

(22) $$k(x_{i},x_{j})=\sigma_{s}\exp(-\frac{1}{2}\sum_{d\;=\;1}^{D}\frac{\left|x_{i}^{\;d}-x_{j}^{\;d}\right|}{l_{d}})$$

The ARD kernel is defined by the signal variance σ_*s*_, the noise variance σ_*n*_ and length-scale parameters *l*_1_, …, *l*_*D*_. The length-scale adjusts the weights of the input data dimensions (e.g., head pose and pupil image position), thus adjusting the relevance of each dimension in predicting the output (e.g., POR coordinates). The kernel function is now specified by the set of hyper-parameters Θ = (σ_*s*_, σ_*n*_, *l*_1_, … *l*_*D*_). It follows that the outcome of future predictions depends highly on the choice of Θ. To obtain a sensible configuration we fitted the hyper-parameters to data 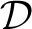 that we collected in a calibration phase. For this, we maximized the marginal log-likelihood given by

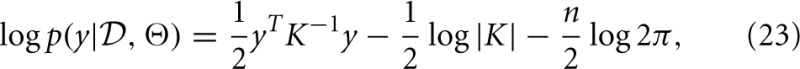



where |*K*| denotes the determinant of *K*. The maximization can be carried out using optimization algorithms such as the conjugate gradient method (Hestenes and Stiefel, 1952).

#### 2.3.2. GPR for gaze-tracking

Given our intention to map the eight-dimensional input data x_*_ = (*h*_*x*_, *h*_*y*_, *h*_*z*_, *h*_ϕ_, *h*_θ_, *h*_ψ_, *p*_*x*_, *p*_*y*_) to screen coordinates *y* = (*u, v*), two Gaussian processes 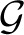
_*u*_, 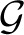
_*v*_ are created for predicting *u* and *v*, respectively. The GP's optimal hyper-parameters Θ_*u*_ and Θ_*v*_ are estimated from a set of calibration data 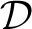 = (*x*_*i*_, *y*_*i*_)^*N*^_*i* = 1_. Details on the calibration procedure are found in section 2.4. We submit 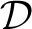
′, a reduced subset of 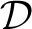, with |
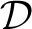
′| ≤ |
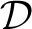
|, for the estimation of the hyper-parameters (Equation 23). We initialize this optimization by setting all parameters to the value of 1. This optimization runs in the range (0, *e*^10^]. After the optimal values for these hyper-parameters are established, the kernel matrices *K*_*u*_ and *K*_*v*_ are computed from the calibration data using Equation (22). This concludes the calibration procedure and 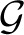
_*u*_, 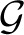
_*v*_ can now be used for predicting the POR. We obtain the target value y_*_ of an input x_*_ by evaluating the respective mean functions μ_*u*_ and μ_*v*_ at x_*_:

(24) $$y_{*}^{T}=\left(\begin{array}{c} u\\ v \end{array}\right)=\left(\begin{array}{c} \mu_{u}(x_{*})\\ \mu_{v}(x_{*}) \end{array}\right)=\left(\begin{array}{c} K_{u}^{*}K_{u}^{-1}y_{u}\\ K_{v}^{*}K_{v}^{-1}y_{v} \end{array}\right)$$

In addition, it is possible to estimate the confidence in each predicted POR by looking at the sample variance

(25) $${\left(\sigma_{*}^{T}\right)}^{2}=\left(\begin{array}{c} \Sigma_{u}(x_{*})\\ \Sigma_{v}(x_{*}) \end{array}\right)=\left(\begin{array}{c} K_{u}^{**}-K_{u}^{*}K_{u}^{-1}K_{u}^{*}{}^{T}\\ K_{v}^{**}-K_{v}^{*}K_{v}^{-1}K_{v}^{*}{}^{T} \end{array}\right).$$

The standard deviation σ_*_ provides an estimate of the predicted POR's reliability. Our implementation makes use of the Gaussian process C++ library libGp^1^. Parameter optimization is performed based on the conjugate gradient implementation in dlib (King, 2009).

### 2.4. Experimental validation

#### 2.4.1. Participants

Twelve participants (age range: 23–35 years; 8 males) with normal or corrected-to-normal vision were recruited for a user evaluation of both mobile gaze-tracking methods. Two participants were authors (Björn Browatzki and Lewis L. Chuang). The remaining 10 participants were employees of the Max Planck Institute for Biological Cybernetics. Their heights ranged from 158 to 193 cm, with a median of 177.5 cm.

#### 2.4.2. Stimuli and apparatus

We recorded eye movements at a sampling rate of 250 Hz, using a head-mounted eye-tracker (EyeLink II, SR Research Ltd). This system required at least one camera to be individually positioned beneath a given eye, so as to capture an image of the pupil in the camera's screen coordinate system.

An infrared optical tracking camera (Advanced Realtime Tracking; 60 Hz) was used to track a fixed configuration of six reflective markers, which were mounted on top of the eye-tracker itself. This provided us with data regarding the user's pose (i.e., head position and orientation) in space. This camera was mounted at the top of the display and oriented to accommodate a large range of user height.

Visual stimuli were displayed on a back-projection screen (1024 by 768 pixels; 220 by 160 cm) with a projector (Christie Mirage S+3K DLP; 120 Hz).

A height-adjustable chin-rest was used in one trial. This was positioned 140 cm away from the screen. From this viewing position, the screen was ±38.2° wide and ±30° high in terms of visual angles.

#### 2.4.3. Procedure

Prior to data collection, the eye-tracking cameras were manually positioned for each participant to provide a clear image of the participant's pupil. To ensure the quality of this camera placement, we performed the calibration and validation procedure provided by SR Research. It should be noted that this procedure did not contribute to the calibration of our mobile gaze-tracking algorithms. In fact, the data collection procedure that follows this was designed to emulate this established calibration–validation process. During this 2 min procedure, participants were required to fixate single dots (0.5°) that were presented one after another on the display. These dots were randomly sampled without replacement from a 3 × 3 grid, which was centered on the display and subtended a field-of-view that approximated ±32° visual angle. This was performed twice. The first time was for calibrating SR Research's algorithm and the second for validating the accuracy of the calibrated algorithm. The cameras were repeatedly re-adjusted until a mean error was achieved that was no larger than 1.5°. We only recorded data from the more accurate eye. Typically, behavioral experiments adopt a mean error threshold of 0.5° prior to recording. However, we adopted a larger error threshold because our chosen eye-tracking system was not intended for use on displays larger than ±16.5°.

Data collection for evaluating our system was performed for three levels of user mobility, which were randomized for their presentation order. We recorded the user's six degree-of-freedom head pose from the motion-tracker and two degree-of-freedom position of one pupil in an eye-tracker's camera image for offline analyses. The participant was either required to restrain his head in a chin-rest (*head fixed*), allowed to move his head freely (*head free*), or allowed to walk freely in a 150 by 145 cm area in front of the display (*walking*).

Each level of user mobility was divided into two phases that differed in terms of their gaze-tracking task. In the first phase (*Dynamic*), the participant was required to fixate a moving red dot on the visual display. This dot moved either vertically or horizontally at a speed of (100 px/s) for at least 100 px, before changing directions randomly, in one of the three alternative cardinal directions. The marker was paused for 750 ms on each change of direction. The overall duration of this phase was 3 min. In the second phase (*Static*), participants fixated red dots that were sequentially presented one after another. The positions of these dots were sampled ten times without replacement from a 5 × 4 grid. This grid was centered in the screen with the dimensions of ±32° width by ±23° height in visual angles. Each of the 20 grid points appeared 10 times in random order for 1500 ms. This resulted in a total of 200 presented stimuli and an overall duration of 5 min.

Short rests were provided to the participants between trials and the full data collection process took approximately 1 h to complete.

### 2.5. Data analysis

The screen coefficients of the geometric algorithm were initially calibrated on the datasets originating from the head-free and walking condition of the first six participants. This is a preliminary step that is necessary only for the geometric method (see section 2.2.3 and Equations 12–15).

Following this, the collected gaze-tracking data were treated to emulate the typical calibration–validation procedure that is performed prior to the use of most video-based eye-trackers (e.g., Eyelink2). First, the data were divided into four datasets for each mobility level. Two datasets were created from the first 2 min and the last minute of the *Dynamic* data collection phase that required participants to fixate a moving target. They are termed *Calib-Dynamic* and *Valid-Dynamic*, respectively. Two more datasets were created from the first 2 min and the last 3 min of stable fixations from the *Static* data collection phase wherein participants sequentially fixated single non-moving stimuli. These are termed *Calib-Static* and *Valid-Static*, respectively. *Calib-Static* and *Valid-Static* were filtered to keep only the stable fixations on the single dots. This was to account for the fact that every user required an undetermined amount of time to saccade toward and maintain a steady fixation on the new target location. Therefore, we removed eye- and head-movements between fixations by ignoring the first 1250 ms of data after each stimulus onset. Only the remaining 250 ms was used to represent the POR for each stimulus.

Three evaluations were performed offline that differed in terms of the pairing between the dataset that was used for training the calibration algorithm and the dataset on which the calibrated algorithm was validated on. These pairings were chosen to exemplify how the data collection procedure could influence the accuracy of the different calibration methods. For the first two evaluations, the regression and geometric calibration methods were trained on *Calib-Dynamic*. Following this, the calibrated algorithms were evaluated in terms of the difference between their estimated PORs on the display, given the datapoints from *Valid-Dynamic* and *Valid-Static*, and the known stimulus position. In a third evaluation, both calibration algorithms were trained on a combined dataset of *Calib-Dynamic* and *Calib-Static* and validated on *Valid-Dynamic* and *Valid-Static*. Neither algorithms was trained on *Calib-Static* alone. This is because the regression method requires a large and variable dataset of eye- and head-movements, which is not available from the discrete and static fixations recorded in *Calib-Static*.

A difference (or error) between the displayed stimulus and the computed POR of either algorithm could be attributed to the given gaze-tracking algorithm and our participants' accuracy in fixating the target stimulus. To allow for comparison to previous methods, these differences were expressed in visual angles rather than pixel distances. Thus, error was computed as the horizontal (azimuth) and vertical (elevation) angular discrepancy between the two direction vectors from either the current position of the participant's head to the estimated POR or to the visual stimulus on the screen. We also report the combined error, which is defined as the angle between these two vectors.

Computation time (measured on a 2.8 GHz desktop CPU) was <3 s for the GP training of the regression method, <1 s for the user specific calibration of the geometric method and <30 s for the calibration of the geometric screen model. If data from the tracking devices can be assumed to be always available, the regression method predicts PORs at approximately 400 Hz on the same hardware. This increases to 3000 Hz if only the POR is computed without its variance. Comparable performance can be achieved by the geometric method. Thus, our methods are computationally efficient and are suitable for real-time applications such as gaze-contingent display changes, given tracking devices with high sampling frequencies and low transmission latencies.

## 3. Results

**Figure 2:**
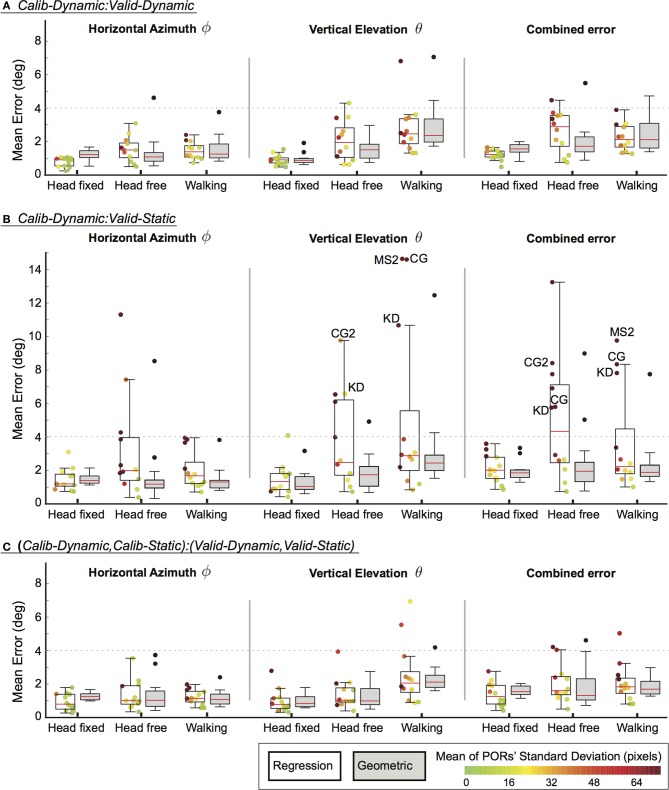
**Mean errors for different calibration–validation dataset pairings (A–C) across the different mobility conditions**. Box-plots represent the median, upper and lower quartile, ±1.5 inter-quartile range and outliers. Data-points for individual participants are plotted for the regression method and their colors correspond to the mean standard deviation of their estimated PORs. Dotted lines are provided to indicate the calibration accuracy of previous work. **(A)** Calib-Dynamic:Valid-Dynamic; **(B)** Calib-Dynamic:Valid-Static; **(C)** (Calib-Dynamic,Calib-Static):(Valid-Dynamic,Valid-Static).

**Figure 3:**
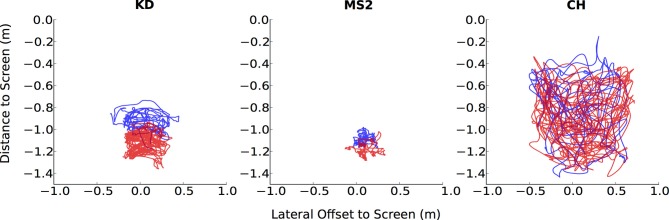
**Individual data for head positions in the walking condition**. Unlike a typical participant (CH), participants with poor gaze-tracking accuracy (KD, MS2) have less overlap between their head positions during Calib-Dynamic 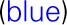 and Valid-Static 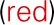.

**Figure 4:**
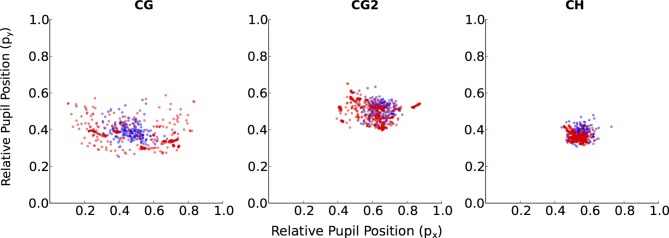
**Individual data for relative pupil positions in the walking condition**. Unlike a typical participant (CH), participants with poor gaze-tracking accuracy (CG, CG2) have pupil positions during Calib-Dynamic 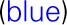 that covers a smaller region than their pupil positions during Valid-Static 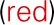.

Three evaluations were performed for different pairings of calibration and validation datasets on the collected datasets of head pose and pupil image data (see section 2.5). These pairings differed in terms of the task that was performed during calibration and validation data collection. The results are plotted separately in Figure 2 for the three user mobility conditions and summarize the mean error for each participant in the horizontal and vertical dimension as well as in the combined visual angle. In addition, the regression method offers a confidence bound for each POR estimate (see section 2.3.2). The mean of these confidence bounds are represented for each participant using a jet-color scheme whereby highly unreliable POR estimates are represented by dark red, which equals a mean standard deviation of 75 pixels and above, while bright green indicates a standard deviation of 0 pixels. The initials of some outlier participant data are highlighted in Figure 2. Their motion- and eye-tracking data are plotted in Figures 3, 4 to understand why the regression method fared poorly for these individuals.

Overall, our geometric method achieved comparable performance to previous work in the head-fixed and head-free conditions. Ronsse et al. (2007) reported a mean absolute error that was less than 3.5° whereas Johnson et al. (2007) reported azimuth and elevation errors that were less than 4.0°. Dotted lines are provided at the 4.0° value in Figure 2 for ease of comparison. Generally, the regression method compares well against the geometric method. Nonetheless, the results of our evaluation highlight some vulnerabilities of the regression method that are addressed in the following paragraphs. Finally, both calibration methods are susceptible to an increase in vertical elevation errors with increasing user mobility.

Task dissimilarity between the calibration and validation phase affected the regression method far more than the geometric method (Figure 2B). This can be remediated by employing similar tasks for calibration and validation (Figure 2A). Alternatively, the calibration algorithm could be trained on gaze behavior that is elicited across multiple tasks (Figure 2C). This would result in more varied data of eye- and head-combinations, which is especially beneficial for training the regression method. Such data need not be exhaustive. Our current example relied on only two tasks that elicited pursuit and fixation gaze behavior, which was sufficiently generalizable.

The regression method appeared to be better than the geometric method for the head-fixed condition, especially for the horizontal azimuth component of estimated PORs (Figures 2A–C). We postulate that the regression method, unlike the geometric method, is able to account for non-linearities caused by large eye-in-head rotations. As mentioned previously, muscle tension in the forehead that result from extreme eye-in-head rotations could cause shifts in the head-mounted eye-tracker. While this would induce inaccuracies in the head-eye-transform model (i.e., 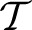) of the geometric method, this will not represent a problem for the regression method as long as such a shift in the eye-tracker is consistently induced.

Differences between the two calibration methods are more apparent when the calibration task varies from the test condition (see Figure 2B). Here, the geometric method generalizes better than the regression method. However, this is not true for all participants. Participants with low gaze-tracking accuracy on the regression method represent outlier data. They are easily identifiable by the large standard deviations (i.e., dark red dots in Figure 2B) in the estimated PORs. If these participants are excluded on this criterion of PORs reliability, the median accuracy of the regression method is comparable (if not superior) to the average accuracy of the geometric method. To reiterate, the geometric method provides no systematic method for removing unreliable data, apart from setting an arbitrarily defined criterion for eye-tracking accuracy during calibration itself.

Four participants demonstrated substantially worse gaze-tracking performance with the regression method, relative to the geometric. Namely, MS, KD, CG, and CG2. These outliers' raw data from the motion- and eye-tracker from the *Calib-Dynamic:Valid-Static* pairing from the *walking* data are respectively plotted in Figures 3, 4, and contrasted against the raw data of participant CH who represented a more typical participant. The main weakness of the regression method is highlighted here in that it requires the calibration data to overlap with the test data that we intend to collect using the calibrated gaze-tracker. Figure 3 shows that MS and KD did not cover as much of the available walking space as CH. As a result, the regression method was not able to accurately generalize from the calibration data to the validation data. The geometric method does not suffer from this problem because it builds a head-eye-transform model (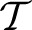) and eye model (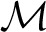) that is independent of the user's position in space. In Figure 4, we note a similar pattern. Participant CG exhibited larger eye-in-head rotations in the *Valid-Static* dataset than her *Calib-Static* dataset. Participant CG2's dataset showed the same, albeit to a lesser extent. In contrast, Participant CH demonstrated an extensive overlap between the eye-tracker data from the calibration and validation datasets.

**Figure 5:**
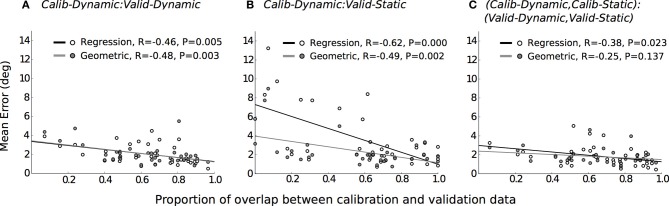
**The relationship between data overlap and mean gaze-tracking error for each evaluation, represented in separate panels**. Each data-point depicts the mean error and its corresponding data overlap for the regression (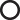) and geometric method (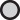). The lines of best fit are plotted for the regression (–) and geometric (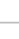) methods, and their coefficient of correlation and significance levels reported. **(A)** Calib-Dynamic:Valid-Dynamic; **(B)** Calib-Dynamic:Valid-Static; **(C)** (Calib-Dynamic,Calib-Static): (Valid-Dynamic,Valid-Static).

Therefore, greater overlaps between calibration and validation datasets should result in higher gaze-tracking accuracy, especially for the regression method. To confirm this, we computed the amount of overlap between the calibration and validation datasets for each of the three evaluations that was performed and examined their relationship to gaze-tracking accuracy (Figure 5). First, a five-dimensional space was defined in terms of head-position (*h*_*x*_, *h*_*y*_, *h*_*z*_) and pupil-position (*p*_*x*_, *p*_*y*_); we omitted head-orientation dimensions because it would have resulted in a large and sparsely populated space. Subsequently, this space was divided into equal-sized bin regions (10 cm for *h*_*x*_, *h*_*y*_, *h*_*z*_; 1500 units for *p*_*x*_, *p*_*y*_) and, for each given evaluation, populated by the calibration and validation dataset. Overlap was defined as the proportion of bin regions that were jointly occupied by calibration and validation datasets to the total number of bin regions occupied by only the validation dataset. This was calculated for each mobility condition per participant, which resulted in 36 data-points per gaze-tracking method for each evaluation. The results are in general agreement with our expectation, there was a significant and weak relationship between dataset overlap and gaze-tracking accuracy for both methods. Figure 5B shows that this relationship was most prominent for the regression method (black line), when the calibration and validation tasks differed from each other. The influence of dataset overlap on gaze-tracking accuracy was considerably reduced for both methods by combining data from the dynamic and static tasks (see Figure 5C).

## 4. Discussion

In this paper, we compared a general geometric method and a regression method for mobile gaze-tracking. Our results indicate that a regression method for gaze-tracking can achieve comparable performance to a geometric approach. Our results also highlight the importance of using an appropriately designed calibration task that is able to elicit variable gaze behaviors. A mobile participant can achieve the same POR by a variety of eye, head and body pose combinations. Thus, submitting a variable and rich data set for calibration can be expected to improve the calibration accuracy of both gaze-tracking methods, especially a regression method. This was similarly noted by Cesqui et al. (2013) who performed calibrations in two phases, first by restraining their participants' heads in order to elicit large eye-in-head rotations and, subsequently, without restraints.

The strength of the geometric method lies in its ability to better generalize across different gaze behavior, regardless of the underlying task. Thus, it was able to maintain reasonable levels of gaze-tracking accuracy even when the calibration task differed from the tested task (Figure 2B). In contrast, the regression method was vulnerable to this difference, presumably because different tasks elicited different patterns of head- and eye-movements in some participants (see Figures 3, 4, respectively). This shortcoming of the regression method could be addressed by ensuring that the calibration data is sufficiently diverse, perhaps by requiring more than one calibration task. In fact, it is generally advisable to calibrate on more than one task, as it is currently shown to benefit both methods (see Figure 5).

Unlike the geometric approach, a regression method for gaze-tracking does not require a specific data input (i.e., eye-in-head rotations) for training. It can be trained on any arbitrary units provided by the eye- and motion-tracking system. Therefore, a regression method can still be used even when the hardware manufacturer does not provide specific information regarding the nature of its available data output.

More importantly, the geometric method has intrinsic limitations that are less easy to overcome. In spite of our repeated efforts in eye-tracker camera placement, the mean accuracy of our eye-tracking calibrations was limited to a range of 0.48° to 1.26°. This is worth mentioning for practical reasons. Under normal circumstances, all of these participants would have been rejected from further participation in the experiment, since most experiments calibrate their participants to an accuracy level of 0.5°. Nonetheless, this level of accuracy in the eye-tracker was to be expected, given the large size of our tested field-of-view, which exceeded the recommended range of the eye-tracker itself (i.e., <±16.5° field-of-view). Under such circumstances, the experimenter faces the dilemma of either relaxing the accuracy threshold for eye-tracker calibration or modifying the experiment. The latter could be achieved by reducing user mobility or the field-of-view. However, this would limit the scope of the researcher's study. The regression approach circumvents this problem in a principled fashion. Recorded PORs can be removed based on the regression method's expressed confidence in their estimation. If this results in a significant proportion, the individual participant's dataset could be removed altogether. Such a process would be transparent, given that the criteria for accepted PORs and proportion of accepted PORs can be reported. Currently, the number of participants who are rejected because of poor eye-tracking calibration are rarely reported, even if the adopted criterion accuracy of 0.5° is fastidiously applied.

The methods reported in this paper do not cover eye-tracking solutions that calibrate and align gaze to the view-frustum of a front-facing video-camera recording (e.g., ETG, Sensoric Instruments GmbH). Such systems allow estimated PORs to be superimposed on a video-recording that approximates a first-person perspective of the user. This approach requires the content of the video-recording to be hand-coded for regions of interest. The methods that we address in this paper estimate PORs according to a known display or world objects without the need for hand-coding. This prevents the researcher from defining the regions of interest in an *ad hoc* fashion.

The accuracy of a geometric method can be improved by defining better models for the underlying eye-head transformation and the pupil's projection to the eye-tracking cameras. Additional procedures could also be introduced to compensate for any errors that might systematically accumulate during experimentation. For example, Cesqui et al. (2013) reported accuracy levels of less than 1° with their mobile gaze-tracking system. This improvement was achieved by introducing a procedure that corrected for drifts due to helmet slippage, by modifying the assumptions for the eye-model and by employing a non-linear optimization algorithm for deriving their calibration parameters. Given the novelty of a regression approach in mobile gaze-tracking, it remains to be seen whether similar improvements can be achieved. Future attempts to improve the regression approach should focus on selecting better algorithms for parameter optimization and improving upon calibration procedures. Unlike a geometric approach, a regression approach does not need to refine the assumptions of the eye-head transformation, eye and physical world model.

The work presented here was conducted to inform researchers who intend to employ gaze-tracking on mobile participants. To this end, we provide software for replicating and improving our methods. The computational efficiency of these methods make them suitable for gaze-contingent experiment designs and applications, if low transmission latencies and synchronization between tracking devices can be ensured (see section 2.5). Based on our results, a regression approach for gaze-tracking approximates the expected accuracy of a geometric approach, if the calibration data captures the effective range of eye and head movements that a user is likely to exhibit in the experiment. In our opinion, a regression approach offers more flexibility and ease of implementation. While the geometric method restricts gaze-tracking accuracy to the limitations of its assumed models and equipment, the regression approach is limited by the design of the calibration task and the employed algorithm. We consider the latter to be more achievable.

## Funding

This research was supported by the Max Planck Society. Part of Heinrich H. Bülthoff's research was supported by the Brain Korea 21 PLUS Program through the National Research Foundation of Korea funded by the Ministry of Education.

### Conflict of interest statement

The authors declare that the research was conducted in the absence of any commercial or financial relationships that could be construed as a potential conflict of interest.

## Footnotes

^1^https://bitbucket.org/mblum/libgp
